# Efficacy of different telemonitoring strategies on chronic heart failure care: A protocol for systematic review and meta-analysis

**DOI:** 10.1097/MD.0000000000028937

**Published:** 2022-04-08

**Authors:** Aichun Wu, Hongbing Li

**Affiliations:** Hemodialysis Room, Wuhan No.1 Hospital, Hubei, China.

**Keywords:** care, heart failure, meta-analysis, mortality, telemonitoring

## Abstract

**Background:**

Growing interest on the effects of telemonitoring on patients with chronic heart failure (CHF) has led to a rise in the number of trials addressing the same or very similar research questions with a concomitant increase in discordant findings. Therefore, we conducted a protocol for systematic review and meta-analysis to compare the effects of different telemonitoring strategies on clinical outcomes in patients with CHF.

**Methods:**

Two individual researchers conducted the platform searches on the PubMed, Cochrane Library, and Embase databases from inception to February 2022. Literature retrieving was carried out through a combined searching of subject terms (“MeSH” on PubMed and “Emtree” on “Embase”) and free terms on the platforms of PubMed and Embase, and through keywords searching on platform of Cochrane Library. Systematic review and meta-analysis of the data will be performed in STATA13.0 software according to the Preferred Reporting Items of Systematic Reviews and Meta-Analysis (PRISMA) guidelines. Two authors independently performed the literature searching, data extraction, and quality evaluation. Risk of bias was assessed using the Cochrane Risk of Bias Tool for randomized controlled trials (RCTs).

**Results:**

The results will be submitted to a peer-reviewed journal.

**Conclusion:**

This meta-analysis will provide a comprehensive analysis and synthesis that can be used as an evidence map to inform practitioners and policy makers about the effectiveness of telemonitoring interventions for patients with CHF.

## 1 Introduction

Chronic heart failure (CHF) is a common problem that is associated with a significant clinical and financial burden.^[1,2]^ Indeed, individuals who experience CHF have higher rates of morbidity and mortality, as well as reduced quality of life.^[3]^ From an economic perspective, estimates from the USA indicate that the cost associated with treating CHF was in excess of 70 billion per year in recent times.^[4]^ With the increase in aging populations worldwide, CHF will remain a significant problem for the foreseeable future, one that will require a concerted effort to optimize management strategies, improve clinical outcomes, and minimize the associated economic burden.

Telemonitoring has been extensively studied as an innovative approach to enable care providers to remotely monitor patients at home and provide timely intervention in the event of clinical deterioration.^[5,6]^ However, the outcomes from individual randomized controlled trials (RCTs) are heterogeneous, with nonsignificant effects obtained in several large and well-designed RCTs.^[7,8]^ Owing to these mixed outcomes, the use of telemonitoring in CHF care has been questioned and has not yet been embraced in clinical recommendations.

Mixed outcomes in telemonitoring studies have been attributed to insufficient support from cardiologists, unsatisfactory patient compliance, low predictive power for clinical deterioration, and improvements in usual care. However, these findings were limited to narrative analyses of individual telemonitoring studies. Several reviews have evaluated specific approaches to CHF care, including structured telephone, videophone, and interactive voice response devices^[9]^; education alone; pharmacist interventions; and clinical support by various care providers.^[10,11]^ These reviews provide valuable insight into the effectiveness of specific types of interventions, but do not explain the mixed outcomes across telemonitoring interventions involving different components of care. Therefore, we conducted a protocol for systematic review and meta-analysis to compare the effects of different telemonitoring strategies on clinical outcomes in patients with CHF.

## 2 Methods

### 2.1 Protocol register

This protocol of systematic review and meta-analysis has been drafted under the guidance of the preferred reporting items for systematic reviews and meta-analyses protocols.^[12]^ It has been registered on open science framework (Registration number: 10.17605/OSF.IO/AWS5C). Ethical approval is not required for this study, as it relies on secondary data.

### 2.2 Search strategy

Two individual researchers conducted the platform searches on the PubMed, Cochrane Library, and Embase databases from inception to February 2022. Literature retrieving was carried out through a combined searching of subject terms (“MeSH” on PubMed and “Emtree” on “Embase”) and free terms on the platforms of PubMed and Embase, and through keywords searching on platform of Cochrane Library. References within included articles were reviewed to include articles that were not included within our literature search. The key terms used for the search were “telemonitoring,” “chronic heart failure,” and “randomize controlled trial.”

### 2.3 Inclusion and exclusion criteria

The study will be selected according to the following criteria:

(1) Participants: adult patients with a diagnosis of CHF;

(2) Intervention measures: the experimental group received telemonitoring strategies;

(3) Control group: the control group only received the usual care;

(4) Outcomes: primary outcomes were all-cause mortality or all-cause hospitalization; secondary outcomes were medical cost and adverse events;

(5) Study type: RCTs.

Articles with no assessment of outcomes mentioned above or no comparison of 2 groups were not included into meta-analysis. Duplicate reports and conference abstracts were excluded. Retrospective studies, letters, case reports or series, biochemical trials, and reviews were also eliminated.

### 2.4 Data extraction

The 2 authors extracted valid data from the included original literature and reviewed the data extracted by each other for accuracy. The data extracted from the studies included title, published year, authors, country, study design, sample size, sex distribution, the type of interventions, and duration of follow-up. Differences and disagreements were settled by discussion; the third authors were consulted if they cannot reach an agreement.

### 2.5 Risk of bias assessment

The methodological qualities of the included studies were assessed independently by the 2 reviewers according to the Cochrane Collaboration's bias evaluation criteria.^[13]^ Its specific contents include the following 6 aspects: the generation of random methods, the allocation of hidden methods, blind methods, lost follow-up and withdrawal, selective reporting results, and other biases. Grading of Recommendations Assessment, Development, and Evaluation (GRADE)^[14]^ was used to assess the quality of evidence, which was classified as high, moderate, low, or very low. Assessments included the risk of bias, inconsistency, indirectness, impreciseness, and other considerations.

### 2.6 Statistical analysis

Two researchers respectively entered the data into the STATA13.0 software. Mean differences (MDs) with a 95% confidence interval (95% CI) were calculated to assess the effect size for continuous outcome data. Risk ratio (RR) with a 95% CI were used as effect size for dichotomous data. Inverse variance method and Mantel-Haenszel analysis method were used for continuous variables and dichotomous variables, respectively. The heterogeneity among the trials was assessed for significance with Q and quantified with *I*^2^. Statistically significant was set at the *P* value < .10. If the studies were homogeneous or the statistical heterogeneity was low, we used the fixed effect-model, although random-effects model was applied when the statistical heterogeneity was moderate or high.

## 3 Discussion

With the rapid development and use of technology, remote patient monitoring, telemonitoring in particular, has become increasingly attractive in the care of ambulatory patients with chronic diseases.^[15,16]^ It offers the promise of frequent review of patients at risk of CHF decompensation while overcoming potential geographical obstacles, and potentially reducing the burden on CHF clinics. Theoretically, CHF hospitalizations could be averted by acting on monitored physiological data before decompensation occurs.^[17]^ The extant literature contains published results from numerous trials investigating the clinical, structural, behavioral, or economic effects of telemonitoring interventions on patients with CHF. However, the evidence based on the utility of telehealth systems to support the management of CHF is inconclusive.

This meta-analysis will provide a comprehensive analysis and synthesis that can be used as an evidence map to inform practitioners and policy makers about the effectiveness of telemonitoring interventions for patients with CHF. Future studies should consider clinical diversity by including subgroup meta-analyses or performing meta-regression, as opposed to pooled meta-analyses.

## Author contributions

**Conceptualization:** Hongbing Li

**Data curation:** Aichun Wu

**Funding acquisition:** Hongbing Li

**Investigation:** Aichun Wu

**Methodology:** Aichun Wu

**Writing - original draft:** Aichun Wu

**Writing - review & editing:** Hongbing Li

**Conceptualization:** Aichun Wu.

**Data curation:** Aichun Wu.

**Funding acquisition:** Hongbing Li.

**Investigation:** Hongbing Li.

**Methodology:** Hongbing Li.

**Writing – original draft:** Aichun Wu.

**Writing – review & editing:** Hongbing Li.

## References

[1] Skrzypek A Mostowik M Szeliga M, et al. Chronic heart failure in the elderly: still a current medical problem. Folia Med Cracov 2018;58:47–56. 30745601

[2] Buckley LF Shah AM. Recent advances in the treatment of chronic heart failure. F1000Res 2019;08. 10.12688/f1000research.20447.1 PMC6944255 31942237

[3] Giallauria F Piccioli L Vitale G Sarullo FM. Exercise training in patients with chronic heart failure: a new challenge for Cardiac Rehabilitation Community. Monaldi Arch Chest Dis 2018;88:987. 30189716 10.4081/monaldi.2018.987

[4] Anker SD Butler J Filippatos GS. Evaluation of the effects of sodium-glucose co-transporter 2 inhibition with empagliflozin on morbidity and mortality in patients with chronic heart failure and a preserved ejection fraction: rationale for and design of the EMPEROR-Preserved Trial. Eur J Heart Fail 2019;21:1279–87. 31523904 10.1002/ejhf.1596

[5] Koike A Sobue Y Kawai M. Safety and feasibility of a telemonitoring-guided exercise program in patients receiving cardiac resynchronization therapy. Ann Noninvasive Electrocardiol 2021;e12926. 34863002 10.1111/anec.12926 PMC8916563

[6] Sardu C Santamaria M Rizzo MR. Telemonitoring in heart failure patients treated by cardiac resynchronisation therapy with defibrillator (CRT-D): the TELECART Study. Int J Clin Pract 2016;70:569–76. 27291327 10.1111/ijcp.12823 PMC5813682

[7] Chaudhry SI Mattera JA Curtis JP. Telemonitoring in patients with heart failure. N Engl J Med 2010;363:2301–9. 21080835 10.1056/NEJMoa1010029 PMC3237394

[8] Koehler F Winkler S Schieber M. Impact of remote telemedical management on mortality and hospitalizations in ambulatory patients with chronic heart failure: the telemedical interventional monitoring in heart failure study. Circulation 2011;123:1873–80. 21444883 10.1161/CIRCULATIONAHA.111.018473

[9] Brinkrolf P Metelmann B Metelmann C. One out of three bystanders of out-of-hospital cardiac arrests shows signs of pathological psychological processing weeks after the incident: results from structured telephone interviews. Scand J Trauma Resusc Emerg Med 2021;29:131. 34496942 10.1186/s13049-021-00945-8 PMC8425096

[10] Romano M. Facilitating supportive care in cardiac intensive care units. Curr Opin Support Palliat Care 2020;14:19–24. 31815767 10.1097/SPC.0000000000000479

[11] Arunmanakul P Chaiyakunapruk N Phrommintikul A, et al. Cost-effectiveness analysis of pharmacist interventions in patients with heart failure in Thailand. J Am Pharm Assoc 2022;62:71–8. 10.1016/j.japh.2021.10.005 34756525

[12] Moher D Shamseer L Clarke M. Preferred reporting items for systematic review and meta-analysis protocols (PRISMA-P) 2015 statement. Syst Rev 2015;4:01. 10.1186/2046-4053-4-1 PMC4320440 25554246

[13] Higgins JP Altman DG Gotzsche PC. The Cochrane Collaboration's tool for assessing risk of bias in randomised trials. BMJ 2011;343:d5928. 22008217 10.1136/bmj.d5928 PMC3196245

[14] Atkins D Eccles M Flottorp S. Systems for grading the quality of evidence and the strength of recommendations I: critical appraisal of existing approaches The GRADE Working Group. BMC Health Serv Res 2004;4:38. 15615589 10.1186/1472-6963-4-38 PMC545647

[15] Ding H Jayasena R Chen SH. The effects of telemonitoring on patient compliance with self-management recommendations and outcomes of the innovative telemonitoring enhanced care program for chronic heart failure: randomized controlled trial. J Med Internet Res 2020;22:e17559. 32673222 10.2196/17559 PMC7381046

[16] Kruse C Pesek B Anderson M, et al. Telemonitoring to manage chronic obstructive pulmonary disease: systematic literature review. JMIR Med Inform 2019;7:e11496. 30892276 10.2196/11496 PMC6446156

[17] Takahashi PY Pecina JL Upatising B. A randomized controlled trial of telemonitoring in older adults with multiple health issues to prevent hospitalizations and emergency department visits. Arch Intern Med 2012;172:773–9. 22507696 10.1001/archinternmed.2012.256 PMC3914200

